# Species-specific and collection method-dependent differences in endometrial susceptibility to seminal plasma-induced RNA degradation

**DOI:** 10.1038/s41598-019-51413-4

**Published:** 2019-10-21

**Authors:** Beatriz Fernandez-Fuertes, José María Sánchez, Sandra Bagés-Arnal, Michael McDonald, Marc Yeste, Pat Lonergan

**Affiliations:** 1 Department of Biology, Faculty of Sciences, Institute of Food and Agricultural Technology, University of Girona, Girona, Spain; 2 School of Agriculture and Food Science, University College Dublin, Belfield, Dublin Ireland

**Keywords:** Reproductive biology, Animal physiology

## Abstract

This study aimed to determine the effect of bull seminal plasma (SP) and sperm on endometrial function. Bovine endometrial explants were incubated with: ejaculated sperm with or without SP, epididymal sperm, or SP alone. Neither ejaculated nor epididymal sperm induced differential expression of *IL1A, IL1B, IL6, IL8, PTGES2, TNFA*, and *LIF*. Interestingly, SP had a detrimental effect on endometrial RNA integrity. Addition of an RNase inactivation reagent to SP blocked this effect, evidencing a role for a SP-RNase. Because bulls deposit the ejaculate in the vagina, we hypothesized that the bovine endometrium is more sensitive to SP-RNase than vaginal and cervical tissues (which come into contact with SP during mating), or to endometrium from intrauterine ejaculators (such as the horse). In addition, due to differences in SP-RNase abundance depending on SP collection method (i.e., with an artificial vagina, AV, or by electroejaculation, EE), this effect was also tested. Bull SP, collected by AV, degrades RNA of mare endometrium, and bovine vagina, cervix and endometrium. However, stallion SP or bull SP collected by EE did not elicit this effect. Thus, results do not support a role for SP in modulating endometrial function to establish pregnancy in cattle.

## Introduction

During semen ejaculation in mammals, epididymal sperm are mixed with secretions of the accessory sex glands, collectively termed seminal plasma (SP). Apart from serving as a vehicle in which sperm are delivered into the female reproductive tract, SP modulates sperm function both *in vivo* and *in vitro* (reviewed by^1^). In addition, there is a growing body of evidence surrounding the role of SP in implantation (reviewed by^2,3^). Much recent work in this area has focused on mice and translation to human reproduction. However, current evidence for a major role of SP in fertility in domestic species, where artificial insemination (AI) in the absence of SP is routine, is relatively weak. Furthermore, embryo transfer (in the absence of exposure of the reproductive tract of the recipient to either sperm or SP) is a routine commercial practice in many livestock species and, under optimal conditions (good quality embryo and appropriate recipient), results in pregnancy rates comparable to those achieved with AI^4,5^.

Nonetheless, evidence exists supporting an important role of SP in fertility in other species. In mice, for example, the absence of SP at mating leads to decreased embryo development in the oviduct and compromised embryo implantation and placental development^6^. In addition, a meta-analysis revealed an increase in human clinical pregnancy rate after SP exposure prior to embryo transfer or near the time of ovum pick-up^7^. These positive effects of SP are thought to be due to its immunoregulatory properties. For example, leukocyte recruitment in female reproductive tissues after mating has been reported in several species^8–12^. Interestingly, in women, condom-protected coitus does not lead to an increase in immune cells in the cervix^9^, suggesting that this phenomenon is not due to mechanical stimulation, but rather to the direct exposure to semen. This notion is supported by literature in mice, where mating of females to males from which seminal vesicle glands (the major contributors to SP in this species) had been excised, also failed to elicit this inflammatory response^12^. In the mouse, SP has been shown to induce antigen-specific expansion of regulatory T cells and tolerogenic dendritic cells, which are important for embryo immune tolerance to paternal antigens during pregnancy^13,14^.

In ruminants, in contrast, there is conflicting evidence on the effect of SP exposure on reproductive function in the female. In cattle, exposing endometrial explants to SP induced changes in the expression of colony stimulating factor 2 (*CSF2*), interleukins -1B, -6, and -17A (*IL1B, IL6, IL17A*), transforming growth factor B1 (*TGFB1*), interferon E (*IFNE*), prostaglandin-endoperoxide synthase 2 (*PTGS2*), and aldo-keto reductase family 1 member C4 (*AKR1C4*)^15^. *In vivo*, infusion of SP into the uterus led to an increase in the expression of *IL1B* and *CSF2* in the horn contralateral to the site of ovulation, while inducing downregulation of *PTGS2* in the ipsilateral horn^15^. However, no differences in pregnancy rates were observed after infusion of SP into the uterus of beef or dairy heifers^16^ or lactating dairy cows^17^, nor when beef or dairy heifers were left with vasectomized bulls for 21 days prior to AI^18^. In addition, although based on small numbers, seminal vesiculectomy in bulls reduced semen volume, and sperm motility and viability, but had no apparent effect on their subsequent fertility^19^. Meanwhile, in sheep, exposure of ewes to SP during estrus induced an increase in neutrophils, and CSF2 and IL-8 secretion in the cervix and uterus, which was not as dramatic when exposed to SP at diestrus^8,20^. In this species, some studies have reported improved pregnancy rates after cervical insemination with frozen-thawed sperm resuspended in SP^21^, while others did not observe such an effect^22^.

It could be argued that because the synepitheliochorial placentation in cattle is not as invasive as the haemochorial placentation in humans or rodents, where the maternal blood comes into direct contact with the fetal chorion, priming of the maternal immune system with paternal antigens may not be as beneficial. However, in the pig, which has a less invasive placenta than cattle and would therefore likely require less immune-modulation, SP induces an endometrial inflammatory reaction and leads to an increase in the average number of viable embryos^10^. This increase is probably due, at least in part, to SP increasing leukocyte infiltration into the ovary, which increases corpus luteum (CL) weight and, therefore, progesterone secretion^23^. In addition, in both pigs and mice, SP exposure at the time of natural mating or AI has also been shown to elicit gene expression changes in the utero-tubal junction and oviduct^6,24,25^, which could impact the development and viability of the early embryo. In the pig, changes in oviductal gene expression are also apparent after insemination with sperm, in the absence of SP^24,26^. Evidence for a role of sperm washed free from SP in modulating the endometrial transcriptome has also been reported in cattle^15^. However, it is not clear whether these changes are due to intrinsic sperm proteins, or to SP proteins acquired at ejaculation. In this latter case, sperm could act as a vehicle of SP components to distal regions of the female reproductive tract.

**Figure 1:**
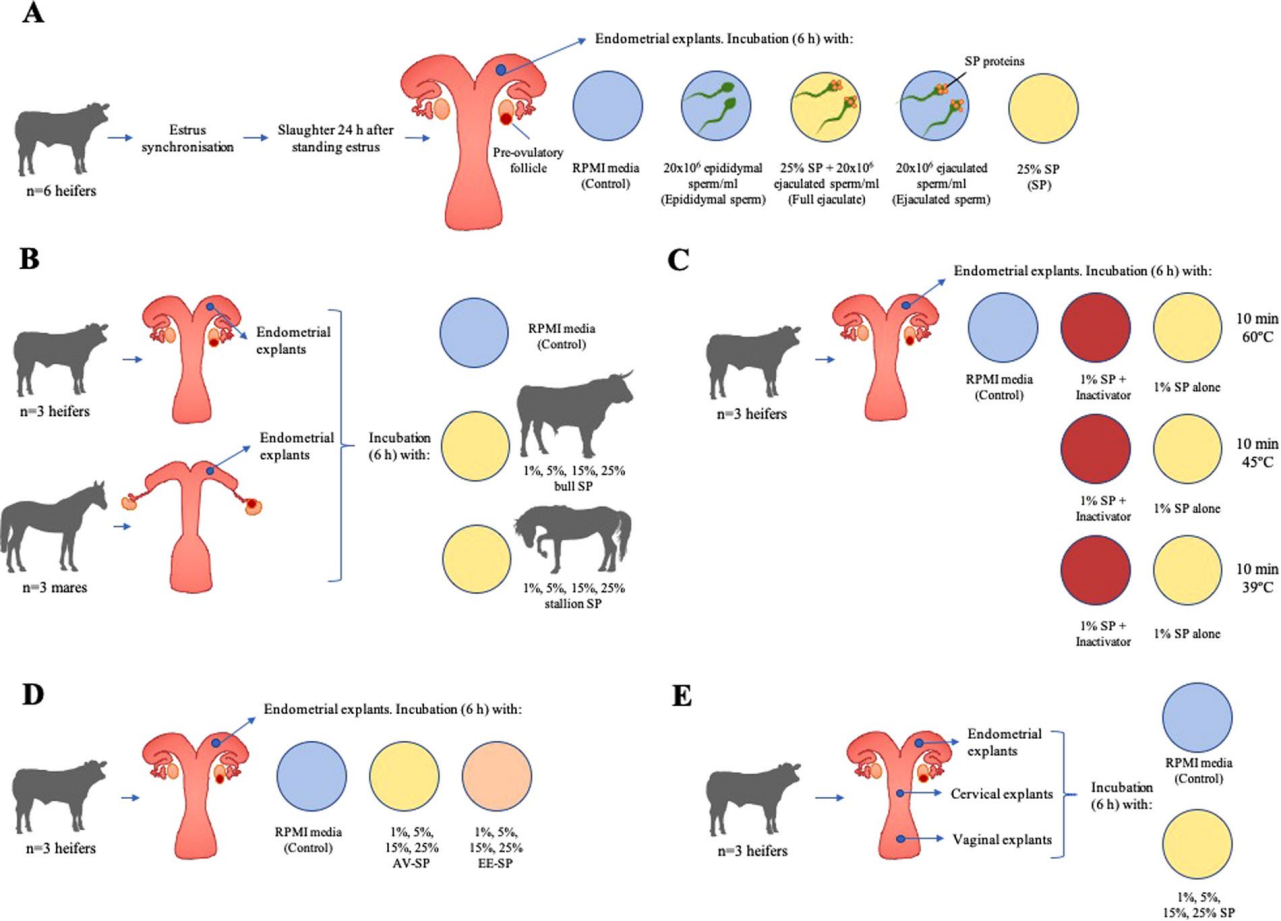
(**A)** Experimental design for Experiment 1. Heifers (n = 6) were estrus synchronized and slaughtered approximately 12 h after being observed in standing estrus. Endometrial explants (n = 5 per animal) were recovered from the ipsilateral horn and incubated for 6 h with: (1) RPMI medium (control), (2) RPMI medium + 5 × 10^6^ epididymal sperm/ml (Epididymal sperm), (3) RPMI medium + 25% SP + 5 × 10^6^ ejaculated sperm/ml (Full ejaculate), (4) RPMI media + 5 × 10^6^ ejaculated sperm/ml (Ejaculated sperm), or (5) RPMI + 25% SP (SP). (**B)** Experimental design for Experiment 2. Bovine (n = 3) and equine (n = 3) reproductive tracts were collected from a commercial slaughterhouse and used to produce endometrial explants (n = 9 per animal). Explants were incubated with 6 h in the presence of RPMI medium alone (control), 1, 5, 15, or 25% bull SP, and 1, 5, 15, 25% stallion SP. (**C)** Experimental design for Experiment 3. Endometrial explants from heifers collected at a commercial slaughterhouse (n = 3) were incubated for 6 h with RPMI media alone (control), or SP which had previously been treated for 10 min at 60 °C, 45 °C or 39 °C with or without a commercial RNase inactivation agent (inactivator). (**D)** Experimental design for Experiment 4. Bovine reproductive tracts (n = 3) collected from a commercial slaughterhouse were used to generate endometrial explants that were incubated (6 h) with RPMI media (control); 1, 5, 15, 25% bull SP collected by artificial vagina (AV-SP); or 1, 5, 15, 25% bull SP collected by electroejaculation (EE-SP). (**E)** Experimental design for Experiment 5. Bovine reproductive tracts (n = 3) were recovered from a commercial abattoir in order to produce endometrial, cervical, and vaginal explants (n = 5 per region and animal). Explants were incubated for 6 h with RPMI media (control) and 1, 5, 15, or 25% bull SP.

Given this background, the aim of the current study was to determine gene expression changes in bovine endometrium following exposure to sperm or SP. To this end, endometrial explants were exposed to different concentrations of SP and ejaculated or epididymal sperm, the latter never exposed to SP. Surprisingly, SP had a very detrimental effect on endometrial RNA quality. We then hypothesized that the bovine endometrium might be more sensitive to the effect of SP, which is naturally deposited into the vagina, than endometrium from intrauterine ejaculator species such as the horse, or to bovine vagina and cervix tissue (which naturally come into contact with SP in cattle). A proteomic analysis of SP from various species identified the presence of a seminal RNase in bull SP, which is not found in stallion, boar, alpaca, camel, ram or buck SP^27^. Thus, to determine whether a SP-RNase was responsible for the effects observed, an RNase inactivation reagent was added to SP prior to endometrial exposure. Furthermore, SP-RNase abundance appears to be dependent on the semen collection method (i.e., with an artificial vagina, AV, or by electroejaculation, EE)^28^, so this effect was also tested. Thus, to better understand the maternal-paternal interaction in the female reproductive tract in cattle we designed a series of crossover approaches using an endometrial explant model (Fig. 1). Such explants maintain normal cellular and extracellular architecture and allow for communication between resident populations of endometrial cells which cannot be achieved using a two-dimensional 2D culture system; in the past, we have used them to interrogate local interaction between the embryo/conceptus and the endometrium^29–31^. The specific objectives of the study were to determine: (i) gene expression changes in bovine endometrium due to exposure to sperm or SP (Experiment 1); (ii) whether SP from an intravaginal ejaculator (bull) or an intrauterine ejaculator (stallion) had a differential effect on endometrial RNA quality, and whether this effect was concentration-dependent (Experiment 2); (iii) whether an RNase inactivation reagent could block the detrimental effect on RNA quality induced by bull SP (Experiment 3); (iv) whether semen collection method alters the effect of SP on the endometrium (Experiment 4): and finally, (v) whether tissues that physiologically (i.e., during natural mating) come in direct contact with bovine SP (i.e., vagina and cervix) exhibit more resilience than the endometrium (Experiment 5).

## Results

### Experiment 1: Effect of bovine sperm or SP on bovine endometrial RNA quality and gene expression

Seminal plasma had a dramatic impact on endometrium RNA integrity, as evidenced by a lower RNA integrity number (RIN) in explants exposed to a complete ejaculate (2.4 ± 0.14, mean ± standard error of the mean, SEM) or SP alone (2.4 ± 0.06), in comparison with the control, epididymal sperm, or ejaculated sperm treatments (6.7 ± 0.43, 6.9 ± 0.32, 6.7 ± 0.30 respectively; p < 0.05). Due to the low RNA quality, those treatments were excluded from further RT-qPCR analysis.

A high variability was found in the relative mRNA abundance of interleukins-1A, -B, -8, and -6 (*IL1A*, *IL1B*, *IL8*, *IL6*), prostaglandin E synthase 2 (*PTGES2*), tumor necrosis factor A (*TNFA*), and leukemia inhibitory factor (*LIF*) between animals, but no effect of treatment (epididymal sperm vs. ejaculated sperm) was observed in comparison with the control (see Supplementary Fig. 1).

### Experiment 2: Effect of bull or stallion SP on RNA quality of bovine or equine endometrium

**Figure 2:**
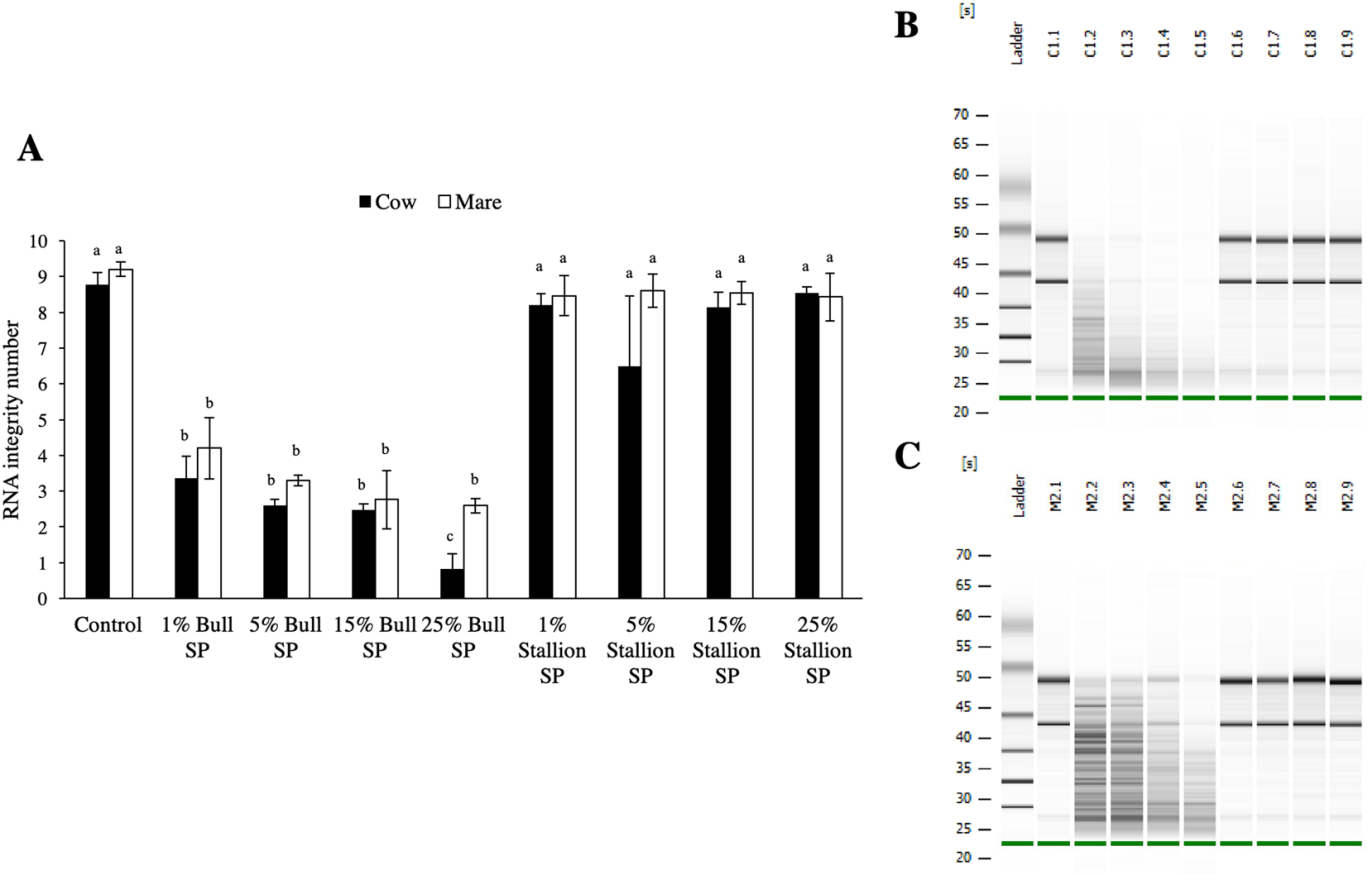
(**A)** RNA integrity number (RIN) of bovine (black) or equine (white) endometrial explants treated with increasing concentrations of bull or stallion seminal plasma (SP). Data reported as mean ± standard error of the mean (SEM). Different superscripts indicate a significant difference *p* < 0.05 (**B)** Electrophoresis gel of endometrial explants from a representative heifer. Lanes C1.1 to C1.9 correspond to: control, 1% bull SP, 5% bull SP, 15% bull SP, 25% bull SP, 1% stallion SP, 5% stallion SP, 15% stallion SP, and 25% stallion SP. Different superscripts indicate a significant difference p < 0.05 (**C)** Electrophoresis gel of endometrial explants from a representative mare. Lanes M2.1 to M2.9 correspond to: control, 1% bull SP, 5% bull SP, 15% bull SP, 25% bull SP, 1% stallion SP, 5% stallion SP, 15% stallion SP, and 25% stallion SP. Note the degradation in RNA quality following exposure of either bovine (C1.2–C1.5) or equine (M2.2–M2.5) endometrium to bull SP compared to the respective controls (C1.1, M2.1, respectively). Gels have been cropped for clarity, for uncropped gels see Supplementary Fig. 2.

Bulls deposit the ejaculate in the anterior vagina, at the external cervical os, as opposed to intrauterine ejaculators (such as the horse). Based on the results of Experiment 1, we hypothesized that (i) bull SP would have a deleterious effect on the RNA quality of bovine but not equine endometrium (which is normally exposed to SP during natural mating) and (ii) stallion SP would not degrade the RNA of either bovine or equine endometrium. After exposure to bull SP, RNA quality of both bovine and equine endometrium dramatically decreased (Fig. 2; electrophoretic gels have been cropped for clarity, for uncropped gels please see Supplementary Fig. 2). In both species, incubation of endometrial explants with as little as 1% bull SP decreased the RIN from 8.8 ± 0.35 (cow control) or 9.2 ± 0.2 (mare control) to 3.4 ± 0.62 (cow) and 4.2 ± 0.85 (mare), respectively (p < 0.05; Fig. 2). Although a SP-concentration effect was observed in both species, equine endometrium RIN did not decrease as much as bovine endometrium RIN when exposed to 25% bull SP (2.6 ± 0.2 vs. 0.8 ± 0.42; p < 0.05).

In contrast, when stallion SP was added to either bovine or equine endometrial explants, no effect on RNA quality was observed (Fig. 2).

### Experiment 3: Effect of pre-incubation of SP with an RNase inactivation reagent on endometrial RNA quality

**Figure 3:**
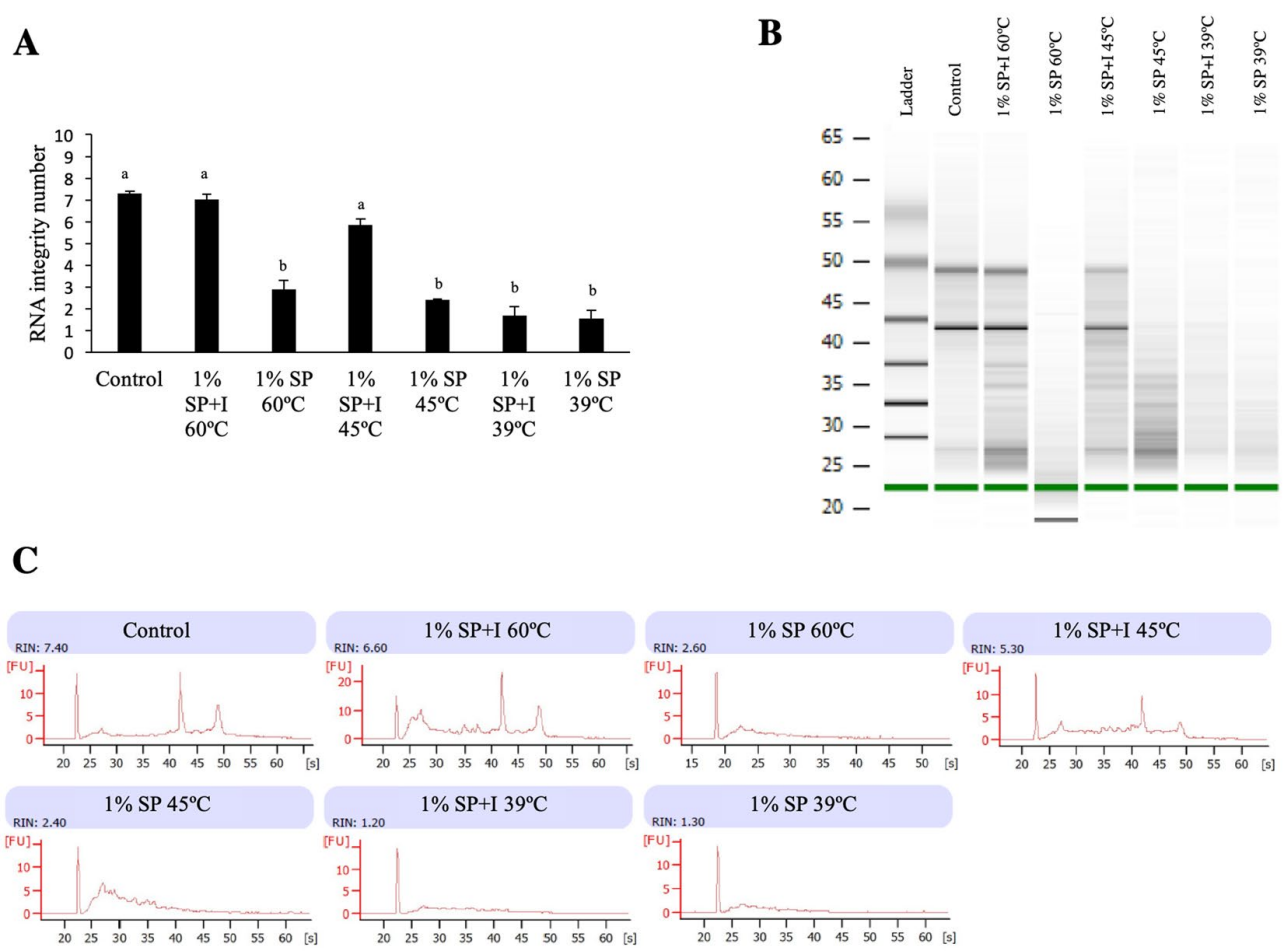
(**A)** RNA integrity number (RIN) of bovine endometrial explants exposed RPMI media alone (control) or to 1% seminal plasma (SP) previously incubated for 10 min at 60 °C, 45 °C or 39 °C with (+I) or without a RNase inactivation reagent. Data reported as mean ± standard error of the mean (SEM). Different superscripts indicate a significant difference p < 0.05. (**B**) Representative electrophoresis gel and electropherograms (**C**) of explants exposed to the indicated treatments. Gel has been cropped for clarity, for uncropped gels see Supplementary Fig. 3.

Given the presence of a seminal RNase in bull SP, which is not present in stallion, boar, alpaca, camel, ram or buck SP^27^, the aim of this experiment was to determine whether the detrimental effects observed following exposure to bovine SP were due to the action of a SP-RNase. When a 1% SP dilution was incubated with an RNase inactivation agent for 10 min prior to endometrial exposure either at 60 °C (the temperature recommended by the manufacturer) or 45 °C (the temperature below which the reagent is inactive), endometrial RNA quality was maintained, as evidenced by a RIN comparable to the blank control explants (7.0 ± 0.23, 5.8 ± 0.29 and 7.3 ± 0.11; p < 0.05; Fig. 3 and Supplementary Fig. 3). In the absence of inactivation reagent, however, RNA quality decreased as in previous experiments, as evidenced by a lower RIN at both 60 °C (2.9 ± 0.41; p < 0.01) and 45 °C (2.4 ± 0.01; p < 0.01). When SP was incubated at 39 °C, a temperature at which the inactivation reagent is not active, explants exposed to this fluid with or without the reagent exhibited an identical drop in RIN compared to the control (1.7 ± 0.42, 1.5 ± 0.39, and 7.3 ± 0.11, respectively; p < 0.01).

### Experiment 4: Effect of semen collection method on SP-induced RNA degradation in bovine endometrium

**Figure 4:**
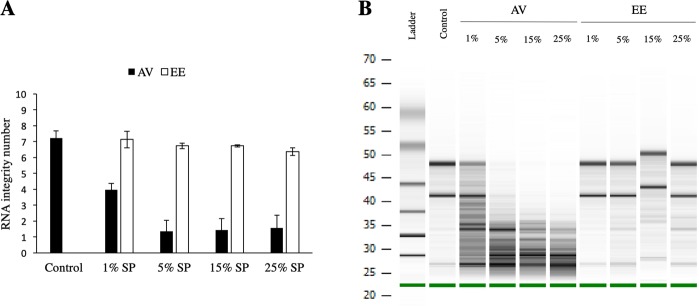
(**A)** RNA integrity number (RIN) of bovine endometrial explants exposed to increasing concentrations (1%, 5%, 15% 25%) of bull seminal plasma (SP) collected by artificial vagina (AV) or electroejaculation (EE). Data reported as mean ± standard error of the mean (SEM). Different superscripts indicate a significant difference p < 0.05. (**B)** Representative electrophoresis gel of explants from endometrial explants exposed to 1%, 5%, 15% 25% SP collected using an AV or by EE. Note the degradation in RNA quality following exposure to increasing concentrations of SP from semen collected with AV and the lack of degradation in EE-derived samples, compared to the control. Gel has been cropped for clarity, for uncropped gels see Supplementary Fig. 4.

Semen collection method has been shown to have an effect on SP composition, including RNase concentration^28^. Thus, this experiment aimed to test whether semen collection method changes the effect of SP exposure on the endometrium. As before, exposure to bull SP collected using an AV resulted in a dose-dependent degradation in RNA quality in bovine endometrial explants. Interestingly, when bovine endometrial explants were exposed to increasing concentrations of SP obtained by EE, no changes in RNA quality were observed (Fig. 4 and Supplementary Fig. 4).

### Experiment 5: Effect of bull SP on RNA quality in different regions of the bovine female reproductive tract

**Figure 5:**
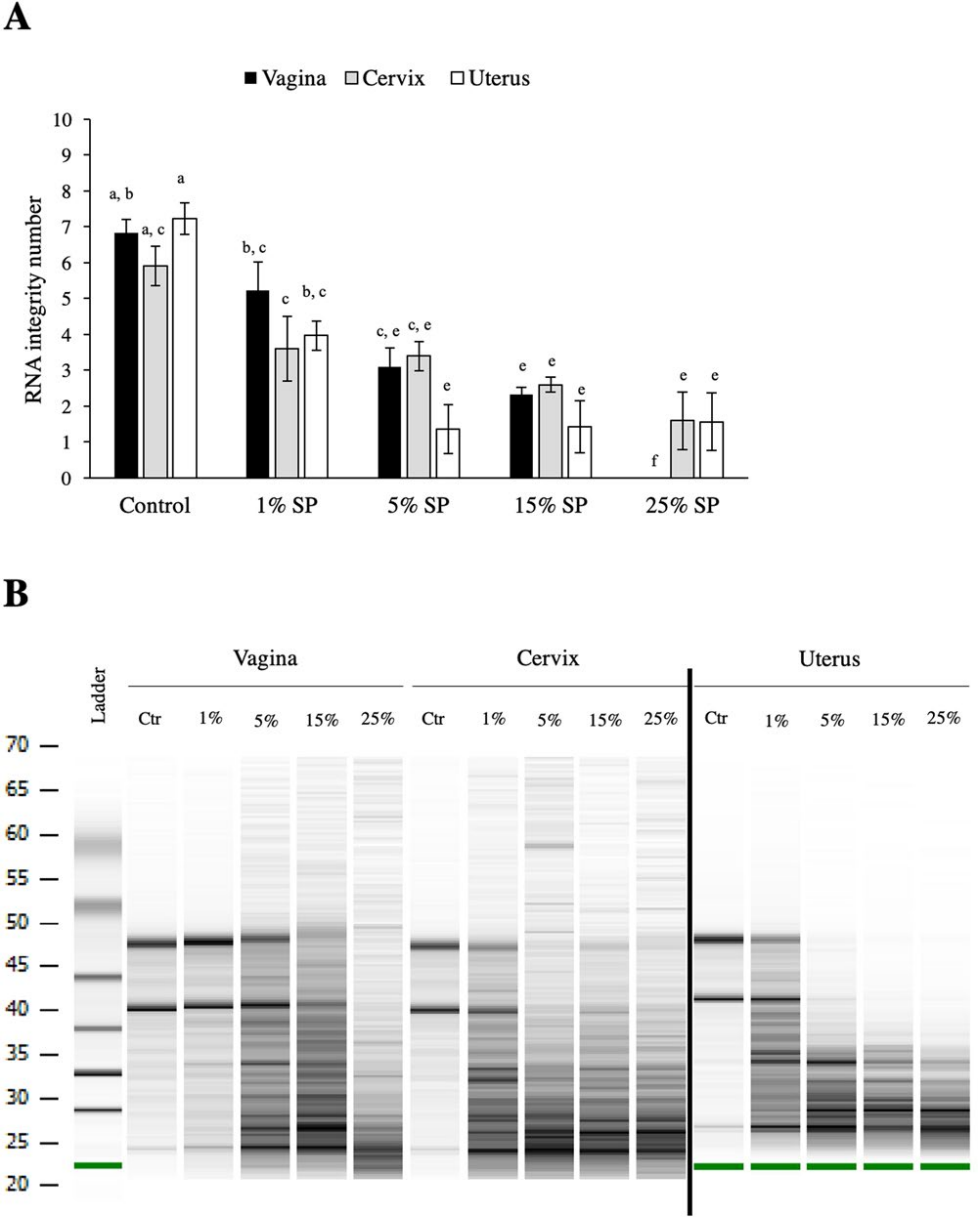
(**A)** RNA integrity number (RIN) of vaginal, cervical or uterine explant treated with increasing concentrations of bull SP. Data reported as mean ± standard error of the mean (SEM). Different superscripts indicate a significant difference p < 0.05. Note the degradation in RNA quality following exposure to increasing concentrations of SP compared to the control. (**B**) Representative electrophoresis gel of bovine vaginal, cervical or endometrial explants exposed to nothing (Control: Ctr.) or 1%, 5%, 15%, 25% bull seminal plasma (SP). This image was created by combining images from two electrophoresis gels, for uncropped, individual gels see Supplementary Fig. 4.

The results of the previous experiments indicate that a bovine SP-RNase has a noxious effect on the endometrium. However, it is likely that very little, if any, SP reaches the endometrium during natural mating in cattle. Thus, it was hypothesized that the cervix and vagina (tissues that come in direct contact with SP during natural mating) would be more resistant to the effect of SP. As opposed to endometrial explants, at low SP concentrations (1%) vaginal and cervical explants had comparable RIN to that of their control treatments (vagina 1% SP: 5.2 ± 0.79 vs vagina control: 6.8 ± 0.37; cervix 1% SP: 3.6 ± 0.9 vs. cervix control: 5.9 ± 0.44; p < 0.05). At SP concentrations of 5% and above all tissues exhibited similar RNA degradation, as evidenced by low RIN values (Fig. 5 and Supplementary Fig. 4). Due to the low RNA yield in the 25% SP vaginal treatment group, no reading was obtained by the Bioanalyzer from any sample from this group.

## Discussion

The main findings of this study are: (1) bovine sperm do not modify the expression of *IL1A, IL1B, IL8, IL6, PTGES2, TNFA*, or *LIF* in the endometrium, irrespective of having had prior exposure to SP, (2) bull SP leads to a concentration-dependent degradation in RNA in both bovine and equine endometrium, as well as in bovine cervix and vagina tissue; (3) stallion SP, or bull SP collected by EE, do not elicit this detrimental effect, even at high concentrations; and (4) incubation of bovine SP with an RNase inactivator reagent prior to co-culture with endometrium abrogates the detrimental effect on RNA quality.

In mice and pigs, there is robust evidence of modulation of the maternal environment through SP which positively impacts embryo survival and development^6,10,12,23,32^. However, similar evidence in cattle is sparse and seemingly contradictory. While differences in endometrial gene expression have been detected after SP infusion into the uterus^15^, no effect on pregnancy outcome was observed in lactating dairy or beef cows after inseminating females that had been exposed to SP in a similar fashion (0.5 mL inseminated 12 h before or at the time of AI^16,17^). Furthermore, no difference in pregnancy rates were observed in beef heifers and cows exposed to a vasectomized bull for 21 days prior to AI compared with control animals with no exposure (42.2% vs 49.5%, respectively^18^), while no difference in reproductive performance of lactating dairy cows bred by timed AI or natural service was reported^33^, suggesting SP does not play a critical role in establishment of pregnancy in cattle. It is important to highlight that the study of Ibrahim *et al*.^15^ was conducted with SP collected by EE, while studies by Odhiambo *et al*.^16^ and Ortiz *et al*.^17^ use SP collected by AV. As discussed below, semen collection method has a major influence on SP composition^28^ which can affect the interpretation of such data.

Differences between species in endometrial response to SP exposure can be due to the differences in type and source of SP proteins, owing to the differences in accessory glands present and the contribution of each gland to the ejaculate^2^. In addition, the site of ejaculate deposition (i.e. intravaginal or intrauterine) likely determines differences in the response of female reproductive tissues to SP. Because of the anatomy of the female reproductive tract and the characteristics of mating in species such as the mouse, pig, and horse, SP comes into direct contact with the uterus^34,35^. In cattle, whether any SP reaches the uterus during natural mating is questionable given the relatively low volume of semen ejaculated (around 5 ml) into the anterior vagina, the length of the cervix, and the high viscosity of cervical mucus at the time of estrus^36,37^. However, at ejaculation, sperm come into contact with SP, leading to proteins binding tightly to the sperm plasma membrane^38^. Experiment 1 tested the hypothesis that sperm might act as a vehicle for SP proteins that could interact with the endometrium once they cross the cervix. To this end, uterine explants were exposed to either epididymal sperm, which have never been in contact with SP, or ejaculated sperm in the presence or absence of SP. Changes in the endometrial expression of *IL1A, IL1B, IL8, IL6, PTGES2, TNFA*, and *LIF* were analyzed by RT-qPCR. All of the genes tested have been shown to regulate the uterine immune response, and their expression is regulated by SP in different species^6,9,10,15,39,40^. In the current study, no differences were observed between epididymal sperm or ejaculated sperm (in the absence of SP) in terms of eliciting endometrial transcriptional changes in *IL1A, IL1B, IL8, IL6, PTGES2, TNFA*, and *LIF*. These results are in agreement with recently published data by Ibrahim *et al*.^15^, where incubation of endometrial explants with sperm alone did not change expression of *CSF2, IL6, CXCL8, IL17A, TGFB1, IFNE, PTGS2, AKR1C4, OXTR* or *IL1B*. However, these authors report that 5% SP (collected by EE), with or without sperm, elicited changes in the relative abundance of these genes (except for *CXCL8* and *OXTR*) in endometrial explants. In the current study, the two treatments containing SP (collected by AV) in Experiment 1 (SP alone and SP + ejaculated sperm) resulted in a high degree of RNA degradation and were therefore excluded from the subsequent qPCR analysis. It could be argued that the concentration of SP used in the Experiment 1 (25%, based on^39^) was too high, as concentrations as low as 4% SP have been shown to negatively impact bovine endometrial cell viability in culture^41^. However, decreasing SP concentration in Experiment 2 to as low as 1% did not overcome the detrimental effect on RNA quality. The 25% SP concentration originally used in Experiment 1 was based on a study in which human ectocervical explants were cultured with 25 or 50% SP^39^, and where no differences in cell viability were observed between these treatments and the control, even after a 24 h incubation^39^. It is interesting to note that humans, as cattle, are intravaginal ejaculators, and the lack of a detrimental effect of SP could be due to the use of cervical explants, a tissue which physiologically comes into direct contact with SP, instead of endometrial explants. Thus, in Experiment 2, we sought to determine whether the negative effect of SP-exposure on endometrial RNA quality was unique to intravaginally ejaculating species (cattle) compared to SP from intrauterine ejaculators (horse), or whether the endometrium from those species exhibited differential sensitivity to SP. Interestingly, both bovine and equine uterine explants exhibited very low RNA quality after exposure to bull SP, but not stallion SP, indicating that it is the bull SP, rather than an over-sensitive endometrium, that elicits these changes.

Comparison of the proteome of SP of various species revealed the presence of the enzyme seminal RNase uniquely in bull SP but not in that of stallion, boar, alpaca, camel, or other ruminants such as the ram or buck^27^. Bovine seminal ribonuclease (BS-RNase) is a close homologue (80% sequence identity) to pancreatic RNase, both members of the secretory RNase A superfamily. This enzyme, produced and secreted by bovine seminal vesicles, is the only known dimeric protein of the ribonuclease superfamily^42^. In addition, it exhibits some unusual biological functions such as selective cytotoxicity towards cancerous cells, and, interestingly, immunosuppression^43^. Indeed, BS-RNase has been shown to inhibit T cell proliferation *in vitro*, without affecting cell viability^44^. The BS-RNase dimer can exist in two distinct quaternary forms. In the major form, designated as MxM, the two monomers interchange the N-terminal helical segment through a linker peptide; in the second form, designated as M = M, this interchange does not occur^42^. While the MxM form shows immunosuppressive, cytotoxic, antiviral and antitumoral properties, the M = M form only has minor antitumoral properties^45^. Both the monomeric and dimeric forms of BS-RNase bind to the plasma membrane of rat fibroblasts and are internalized through endosomes^46^; however, although the monomeric form is more active than the dimeric enzyme, it is not cytotoxic^47^. The different biological properties of the monomeric and the two dimeric forms are due to the ability of the MXM form to evade the cytosolic RNase inhibitor (RI)^48^. In the cytosol, the RI binds and inhibits the monomeric BS-RNase, but cannot bind to the dimeric forms. In the reducing environment of the cytosol, M = M dissociates into monomers, while the N-terminal swap of the non-covalent dimer is sufficient to stabilize the MxM form, allowing it to evade the RI^48,49^. Despite this information, the mechanisms through which BS-RNase acts as an immunosuppressive agent are not clear.

To confirm that BS-RNase was responsible for the degradation of RNA quality observed, in Experiment 3, bull SP was treated with an RNA inactivator reagent prior to its addition to the endometrial explants. The use of this non-enzymatic reagent was chosen because, as mentioned above, the dimeric form of BS-RNase allows it to evade protein-based inhibitors. According to the manufacturer’s instructions, the RNAsecure™ reagent should be incubated with the solution to be treated for 10 min at 60 °C, but the reagent is active above 45 °C. Because of the risk of SP protein denaturation due to incubation at 60 °C (irrespective of inactivation reagent activity), we included a control group where SP in the absence of the inactivation reagent was incubated for 10 min at 60 °C, and we also tested a lower temperature of 45 °C and 39 °C (at which the reagent should show no activity). As expected, endometrial RNA quality of explants incubated in the presence of SP previously treated with RNAsecure™ reagent at 60 °C or 45 °C did not differ to that of the control, whereas SP exposed to the same temperatures in the absence of inactivation reagent dramatically reduced endometrial RIN. These low RIN values were comparable to those observed in explants exposed to SP previously treated at 39 °C, a temperature at which the reagent has no activity, regardless of the presence of the inactivation reagent. This demonstrates that a SP-RNase, likely BS-RNase, is responsible for the RNA degradation observed in prior experiments. Further evidence for the central role of BS-RNase in endometrial response to SP comes from the comparison of the effect of semen collected with an AV (i.e., equivalent to natural ejaculation), with that collected by EE on endometrial RNA integrity. Exposure of uterine explants to SP from ejaculates collected by EE did not lead to degradation of RNA, as observed with SP from AV-collected bulls. Seminal plasma protein composition has been shown to differ between EE and AV collection in Brahman bulls^28^, which is likely due to differences in accessory gland stimulation (and thus differences in the volume secreted) between both collection methods. It is of great interest that, in that study, BS-RNase was 15-fold more abundant in SP collected by AV than EE^28^. This could explain the lack of SP toxicity that Ibrahim *et al*.^15^ reported in their study, as they incubated explants in 5% SP collected by EE.

Since cattle ejaculate intravaginally, the aim of Experiment 5 was to determine whether more proximal regions of the cow reproductive tract are more resilient to SP, and the effects of BS-RNase, than the endometrium. Both cervical and vaginal explants exposed to the lowest SP concentration (1%) had comparable RNA quality to their untreated controls, suggesting a lower sensitivity to the detrimental effect of SP than the endometrial explants. Although statistically significant, a RIN value of 3.6 (the average value for cervix explants exposed to 1% SP) indicates a high level of RNA degradation. RNA degradation increased when concentrations of 5% or greater were used, with RIN values comparable to endometrial RIN values in those treatments. It is important to note that*, in vivo*, only the surface epithelia come into contact with SP, whereas in the explant model, the whole tissue is surrounded by this fluid. Throughout the 6 h culture the cervix and vagina explants seemed to continue to produce a thick mucus (not quantified), characteristic of the estrous cycle stage. It is possible that this mucus protects the epithelia from the noxious effects of SP *in vivo*, and that the amount produced was sufficient to preserve the tissue at the lowest SP concentration. In mouse and human cell cultures, there is evidence for cells exhibiting differences in their ability to inhibit RNA degradation by BS-RNase^46,50^; although BS-RNase was internalized into both normal and cancerous fibroblasts *in vitro*, it only reached the cytosol and degraded RNA of malignant cells^46^. It is not clear what mechanisms inhibit BS-RNase from reaching the cytosol in non-cancerous cells or why it would degrade endometrial RNA as occurred in this study. However, in addition to the mechanical barrier provided by mucus, bovine vaginal and cervical epithelial cells may have the ability to block BS-RNase activity.

In mice, transforming growth factor-β (TGF-β) has been shown to be the principal stimulating agent of the SP-induced inflammatory response of the uterus^51^. However, the SP factor(s) responsible for the changes observed in cattle is still not clear. BS-RNase, with its immunoregulatory and antitumoral properties might play an important role in modulating the bovine female reproductive tract. In addition, it is interesting to note that this protein is overexpressed in SP from high- compared to low-fertility dairy bulls^52^. However, key aspects such as how this immunoregulatory role is elicited, or how the vaginal and cervical epithelia might evade the cytotoxic effect of this enzyme *in vivo* remain to be elucidated.

In conclusion, the results of this study do not support a role for SP in modulating bovine endometrial function in order to establish pregnancy in cattle. However, as bulls ejaculate intravaginally, we cannot rule out the possibility that SP might have an indirect effect on the uterine environment by eliciting a response in the vagina and cervix that might propagate to this region. Experiments using natural mating in cattle, rather than SP infusion into the uterus as has been carried out by others^15–17^, would provide evidence on whether changes in distal regions of the reproductive tract are present after mating. Published literature indicates that SP exposure in cattle does not lead to an increase in pregnancy rates^16–18^. However, it is important to note that, in mice, SP exposure not only has an impact on embryo viability, but also on embryo quality and subsequent offspring phenotype^6,53^. This effect is probably due to the changes elicited in the oviductal and uterine environments. Whether this scenario might be true for the bovine remains unknown and should be addressed in future studies. The dramatic effects of bull SP on RNA quality were surprising. The gene coding for BS-RNase, the most likely candidate behind this effect, underwent an episode of rapid sequence evolution in modern cattle^54^, which suggests an immunological function, supporting the results observed in *in vitro* cell culture studies^44,55,56^. Understanding the role that this enzyme plays in the female reproductive tract and its regulation will provide new insights into the interaction between SP and the female reproductive tract, and the immune regulation that is established in this region to both destroy pathogens while allowing embryo development. Finally, results highlight the significant impact of method of semen collection (AV vs EE) on studies examining the influence of SP on endometrial function and the need for caution when interpreting data in the literature.

## Materials and Methods

All experimental procedures involving animals were approved by the Animal Research Ethics Committee of University College Dublin and licensed by the Health Products Regulatory Authority (HPRA), Ireland, in accordance with Statutory Instrument No. 543 of 2012 (under Directive 2010/63/EU on the Protection of Animals used for Scientific Purposes).

### Reagents

Unless otherwise stated, all chemicals were sourced from Sigma-Aldrich (Dublin, Ireland).

### Experiment 1: Effect of bovine sperm or SP on bovine endometrial RNA quality and gene expression

The aim of this experiment was to determine gene expression changes induced in the bovine endometrium due to exposure to sperm or SP. The experimental design is summarized in Fig. 1A.

#### Generation of endometrial explants

The estrous cycles of six cross-bred beef heifers were synchronized using an 8-day intravaginal progesterone device (PRID® Delta, 1.55 g progesterone, Ceva Santé Animale, Libourne, France), together with a 2 ml intra muscular injection of a synthetic gonadotrophin releasing hormone (Ovarelin®, equivalent to 100 µg Gonadorelin, Ceva Santé Animale) administered on the day of PRID insertion. One day prior to PRID removal, all heifers received a 5 ml intra muscular injection of prostaglandin F2 alpha (Enzaprost®, equivalent to 25 mg of Dinoprost, Ceva Santé Animale) to induce luteolysis. Heifers were slaughtered at a commercial abattoir approximately 24 h after standing estrus (Day 0), and just prior to the expected ovulation (which occurs ∼28 h after the onset of estrus^57–59^). Reproductive tracts were collected, placed on ice and transported to the laboratory within approximately 2 h of slaughter. Uteri were processed as described previously^60^. Briefly, the uterine horn ipsilateral to the ovary containing the pre-ovulatory follicle was opened longitudinally with sterile scissors. The exposed endometrium was washed with Dulbecco’s phosphate-buffered saline solution (PBS) supplemented with 1% Antibiotic-Antimycotic (ABAM; Gibco, ThermoFisher Scientific, Dublin, Ireland), and tissue samples were obtained from intercaruncular areas with the use of a sterile 8 mm biopsy punch (Stiefel Laboratories Ltd, High Wycome, UK). Sterile blades were used to dissect the endometrium away from the myometrium. Once dissected, the explants were washed twice in Hank’s balanced salt solution (Gibco, ThermoFisher Scientific) containing 1% ABAM, and then transferred into a 24-well plate, so that each well contained one explant in 1 ml Roswell Park Memorial Institute-1640 (RPMI) medium (Gibco, ThermoFisher Scientific) supplemented with 1% ABAM. Explants were cultured endometrial side up at 39 °C under an atmosphere of 5% CO_2_ for 4 h before use.

#### Preparation of sperm and SP

To obtain epididymal sperm, testes from 3 bulls were collected from a local abattoir and transported within 2 h to the laboratory at ambient temperature. A small incision was made in the cauda epididymis and the lumen of the deferent duct was cannulated with a blunted 22 G needle. Sperm cells were then gently flushed through the cauda with a 5 ml syringe loaded with PBS at 38 °C, as described by Druart *et al*.^61^. Epididymal sperm recovered from the 3 bulls were pooled and washed through a 90–45% discontinuous Percoll gradient (Pharmacia, Uppsala, Sweden) for 9 min at 700 g, followed by a second centrifugation in RPMI medium at 200 g for 5 min. Sperm concentration was assessed with a hemocytometer and adjusted to 5 × 10^6^ sperm/ml.

To obtain fresh sperm and SP, ejaculates from 3 Holstein Friesian bulls collected at a local AI centre using an AV were pooled. One ml of the pooled semen was washed through a Percoll gradient as described above, to obtain ejaculated sperm free of SP. After a second wash in RPMI medium, sperm concentration was adjusted to 5 × 10^6^ sperm/ml either in RPMI medium + 25% SP (designated as “full ejaculate”) or RPMI medium alone (designated as “ejaculated sperm”). SP was obtained by centrifugation of the rest of the pooled ejaculates (9 min at 700 g). To ensure the absence of sperm, the SP was then filtered through a 0.5 µm pore filter (Sarstedt, Wexford, Ireland).

#### Treatment of uterine explants

After 4 h equilibration in RPMI medium, explants were transferred to wells containing: (1) RPMI medium alone (Control), (2) RPMI medium + 5 × 10^6^ epididymal sperm/ml (Epididymal sperm), (3) RPMI medium + 25% SP + 5 × 10^6^ ejaculated sperm/ml (Full ejaculate), (4) RPMI media + 5 × 10^6^ ejaculated sperm/ml (Ejaculated sperm), (5) RPMI + 25% SP (SP). The decision to add 25% SP was based on the work of Introini *et al*.^39^, who incubated human cervical explants for 12 h with this concentration of SP and reported no differences in apoptosis compared to a control. Explants were incubated with the five treatments for 6 h at 39 °C under an atmosphere of 5% CO_2_ after which they were washed twice in RPMI media to remove sperm, snap frozen, and stored at −80 °C until further use. Six replicates were carried out, each replicate corresponding to a synchronized heifer (n = 6).

#### RNA extraction and cDNA synthesis

Total RNA extraction was carried out as described previously^62^. Briefly, total RNA was extracted from ~50 mg of endometrial explants using Trizol reagent (Invitrogen, Carlsbad, CA) per the manufacturer’s instructions. On-column DNase digestion and RNA clean-up was performed using the Qiagen mini kit (Qiagen). The quantity of RNA was determined using the Nano Drop 1000 (Thermo Fisher Scientific), whereas quality was assessed with the Agilent Bioanalyzer (Agilent Technologies). The Agilent Bioanalyzer performs RNA electrophoresis to evaluate RNA degradation and provides an RNA Integrity Number (RIN) for each sample. The process of RNA degradation is gradual and, as it proceeds, there is a decrease in the 18S to 28S ribosomal band ratio and an increase in the baseline signal between the two ribosomal peaks and the lower marker. The RIN allows the classification of eukaryotic total RNA based on a numbering system from 1 to 10, with 1 being the most degraded profile and 10 being the most intact.

For each sample, cDNA was prepared from 500 ng of total RNA using the High Capacity cDNA Reverse Transcription Kit (Thermo Fisher Scientific) according to the manufacturer’s instructions. The purified cDNA was then diluted in RNase- and DNase-free water up to a volume of 300 µL and stored at −20 °C for subsequent analysis.

#### Quantitative real-time PCR Analysis

Quantitative real-time PCR (qPCR) was used to investigate changes in endometrial gene expression due to treatment and was carried out as described previously^63^. All primers were designed using Primer Blast software (https://www.ncbi.nlm.nih.gov/tools/primer-blast/) (Supplementary Table 1). Briefly, duplicate qPCR assays were performed in a total volume of 20 µL, containing 10 µL FastStart Universal SYBR Green Master (Roche Diagnostics Ltd., West Sussex, UK), 1.2 µL forward and reverse primer mix (300 nM final concentration), 2.6 µL nuclease-free water and 5 µL cDNA template on the ABI Prism 7500 Fast Sequence Detection System (Life Technologies). Thermo-cycling conditions were as follows: 95 °C for 10 min for one cycle, followed by 95 °C for 15 s, and 60 °C for 1 min for 40 cycles. A dissociation curve was also added to ensure specificity of amplification. The presence of a single sharp peak in the melt curve analysis confirmed the specificity of all targets. A total of eight potential reference genes (*GAPDH, ACTB, RPL18, PPIA, YWHAZ, RNF11, H3F3A, SDHA*) were analyzed using the geNorm function with the qbase + package (Biogazelle, Zwijnaarde, Belgium) to identify the best reference genes^64^. Because they were more stably expressed (average geNorm M ≤ 0.5), the reference genes selected were: *ACTB* and *PPIA*. A standard curve was included for each gene of interest as well as for the reference genes to confirm primer efficiencies for all targets were between 90% and 110%. The threshold cycle (Ct) for each sample was automatically calculated using the default settings within the SDS software (SDS 1.4, ABI).

### Experiment 2: Effect of bull or stallion SP on RNA quality of bovine or equine endometrium

Based on the results of Experiment 1, the aim of this experiment was to determine whether SP from an intravaginal ejaculator (bull) or an intrauterine ejaculator (stallion) had a differential effect on endometrium RNA quality, and whether this effect was concentration-dependent. The experimental design is summarized in Fig. 1B. To this end, 3 bovine and 3 equine female reproductive tracts were collected from commercial abattoirs. Bovine tracts were chosen based on the presence of a large dominant follicle and a regressing CL, while mare tracts presented a large dominant follicle and no visible CL, both bovine and mare tracts presented thick mucus in the vagina and cervix indicating the proximity to estrus. Nine explants were prepared from each uterus following the protocol described above.

#### Preparation of SP

Fresh ejaculates collected using an AV from 3 different bulls were pooled and processed as described above to obtain sperm free-SP. A fresh ejaculate from one stallion was processed following the same protocol. In both cases, SP concentration was adjusted to either 1%, 5%, 15% or 25% in RPMI medium.

#### Treatment of endometrial explants

After 4 h equilibration in RPMI medium, bovine and equine endometrial explants were transferred individually to wells containing the following treatments: (1) RPMI medium alone (bovine control) (2) RPMI medium alone (equine control), (3) 1% bull SP, (4) 5% bull SP, (5) 15% bull SP, (6) 25% bull SP, (7) 1% stallion SP, (8) 5% stallion SP, (9) 15% stallion SP, and (10) 25% stallion SP. Explants were incubated for 6 h at 39 °C under an atmosphere of 5% CO_2_. After incubation, explants were washed twice in RPMI medium, snap frozen, and stored at −80 °C until further use. RNA extraction and quality assessment were carried out as described above.

### Experiment 3: Effect of pre-incubation of SP with an RNase inactivation reagent on endometrial RNA quality

In order to confirm that the deleterious effect of SP exposure on bovine endometrial RNA quality was due to endogenous RNase, SP was incubated in the presence of an RNase inactivation agent (Ambion® RNAsecure™, Invitrogen) prior to its addition to endometrial explants. This reagent, which is commercialized at 25x concentration, was diluted in 1% SP (in RPMI media) to the 1x working concentration. According to the manufacturer’s instructions, this non-enzymatic reagent irreversibly inactivates RNases when incubation takes place at 60 °C for 10 min, although RNAsecure™ reagent is active above 45 °C. Thus, the following treatment groups were assessed: (1) RPMI medium alone (blank control), (2) 1% SP incubated for 10 min at 60 °C + inactivation reagent, (3) 1% SP incubated for 10 min at 60 °C, (4) 1% SP incubated for 10 min at 4 5 °C + inactivation reagent, (5) 1% SP incubated for 10 min at 45 °C, (6) 1% SP incubated for 10 min at 39 °C + inactivation reagent, (7) 1% SP incubated for 10 min at 39 °C. The experimental design is summarized in Fig. 1C. Explants were incubated with these treatments for 6 h under the conditions described above. After incubation, explants were washed twice in RPMI media, snap frozen and stored at −80 °C prior to RNA quality assessment.

### Experiment 4: Effect of semen collection method on SP-induced RNA degradation in bovine endometrium

The aim of this experiment was to determine whether collection method (i.e. EE or AV) affects SP-induced RNA degradation in the bovine endometrium. The experimental design is summarized in Fig. 1D. Fresh ejaculates were collected by AV from 3 bulls and pooled. Ejaculates from 3 other bulls were collected by electroejaculation (EE) and pooled. Pooled ejaculates were processed as described above to generate sperm-free SP samples. Bovine reproductive tracts at the pre-ovulatory stage were collected from 3 animals as described above, and 9 endometrial explants were obtained from each tract. Treatment groups for this experiment were as follows: (1) RPMI medium alone (blank control), (2) 1% AV SP, (3) 5% AV SP, (4) 15% AV SP, (5) 25% AV SP, (6) 1% EE SP, (7) 5% EE SP, (8) 15% EE SP, (9) 25% EE SP. Explants were incubated with these treatments for 6 h under the conditions described above. After incubation, explants were washed twice in RPMI media, snap frozen and stored at −80 °C until RNA extraction.

### Experiment 5: Effect of bull SP on RNA quality in different regions of the bovine female reproductive tract

The aim of this experiment was to compare the effect of bull SP on vaginal and cervical tissue – both of which come in direct contact with SP during natural mating – and on endometrial tissue. The experimental design is summarized in Fig. 1E. Three bovine reproductive tracts at the pre-ovulatory stage were collected at the abattoir as described above. Explants were prepared from the vagina, distal cervix and uterus of each animal (n = 5 explants per region per animal) as described above. Bull SP was processed as described above. For this experiment, 15 treatments were assessed: (1) vagina + RPMI medium (vagina control), (2) vagina + 1% SP, (3) vagina + 5% SP, (4) vagina + 15% SP, (5) vagina + 25% SP, (6) cervix + RPMI medium (cervix control), (7) cervix + 1% SP, (8) cervix + 5% SP, (9) cervix + 15% SP, (10) cervix + 25% SP, (11) endometrium + RPMI medium (endometrium control), (12) endometrium + 1% SP, (13) endometrium + 5% SP, (14) endometrium + 15% SP, and (15) endometrium + 25% SP. Explants were incubated with these treatments for 6 h and processed as described above. RNA extraction and quality assessment were carried out as already described.

### Statistical analysis

For the qPCR data in Experiment 1, Ct values were imported into the qbase + analysis package. Data were normalized using the geometric mean of the reference genes as identified by geNorm. Relative expression values were automatically calculated by the software using a modified version of delta-delta Ct method (∆∆Cq; also known as∆∆CT)^65^. One-way ANOVA was performed on the log transformed data.

For all other experiments, results were analyzed using a statistical package (IBM SPSS for Windows 25.0). Normality and homogeneity of variances were tested through Shapiro-Wilk and Levene tests, respectively. In all cases, RIN was the variable. In Experiments 1, 3 and 4, one-way ANOVA (factor: treatment) followed by post-hoc Sidak test was run. In Experiment 2, data were analyzed with a two-way ANOVA (factors: treatment; female: cow vs. mare). The level of significance was set at p < 0.05.

## Supplementary information

Dataset 1
