# Supplementary material for: Species-specific and collection method-dependent differences in endometrial susceptibility to seminal plasma-induced RNA degradation
Source: Sci Rep. 2019 Oct 21;9:15072. doi: 10.1038/s41598-019-51413-4 (PMC6803643; doi:10.1038/s41598-019-51413-4)

SUPPLEMENTARY DATA

Species-specific and collection method-dependent differences in endometrial susceptibility to seminal plasma-induced RNA degradation

Beatriz Fernandez-Fuertes[1]*, José María Sánchez [2], Sandra Bagés-Arnal[2], Michael McDonald[2], Marc Yeste[1], Pat Lonergan[2]

[1]Department of Biology, Faculty of Sciences, Institute of Food and Agricultural Technology, University of Girona, Girona, Spain; [2]School of Agriculture and Food Science, University College Dublin, Belfield, Dublin, Ireland

Supplementary Table 1. Gene symbol, accession number, primers sequence and amplicon size for all genes analysed by quantitative real-time PCR.

Gene symbol	Accession number	Primer sequence (5´-3)	Amplicon size
IL1A	NM_174092.1	TTCGAGATATGTCAGGTCCATACC<br>AGTCACAGGAAGCTGAGAATCC	121
IL1B	NM_174093.1	CTTCATTGCCCAGGTTTCTG<br>CAGGTGTTGGATGCAGCTCT	142
IL6	NM_173923.2	ACGAGTATGAGGGAAATCAGGA<br>CAGTGTTTGTGGCTGGAGTG	117
IL8	NM_173925.2	GACTTCCAAGCTGGCTGTTG<br>GGGTGGAAAGGTGTGGAATG	135
PTGES2	XM_010813540.2	CGTGAAAGGCTGTCCCTTTA<br>ATCTAGTCCAGAGTGGGAAGAG	97
TNFA	EU276079	CTCTTCTGCCTGCTGCACTTC<br>CCATGAGGGCATTGGCATACG	102
LIF	NM_173931.1	CCAGCTGGGACAACTCAACA<br>CGGGAAGTCAGTCACGTTGG	121

Supplementary Fig. 1 Relative quantity of interleukins -1A, -1B, -6, and -8 (*IL1A, IL1B, IL6, IL8*), prostaglandin-endoperoxide synthase 2 (*PTGS2*), tumor necrosis factor A (*TNFA*), and leukemia inhibitory factor (*LIF*) in bovine endometrial explants exposed to RPMI media alone (control), ejaculated sperm (Ejac. Sperm) or epididymal sperm (Epid. Sperm) for 6 h.

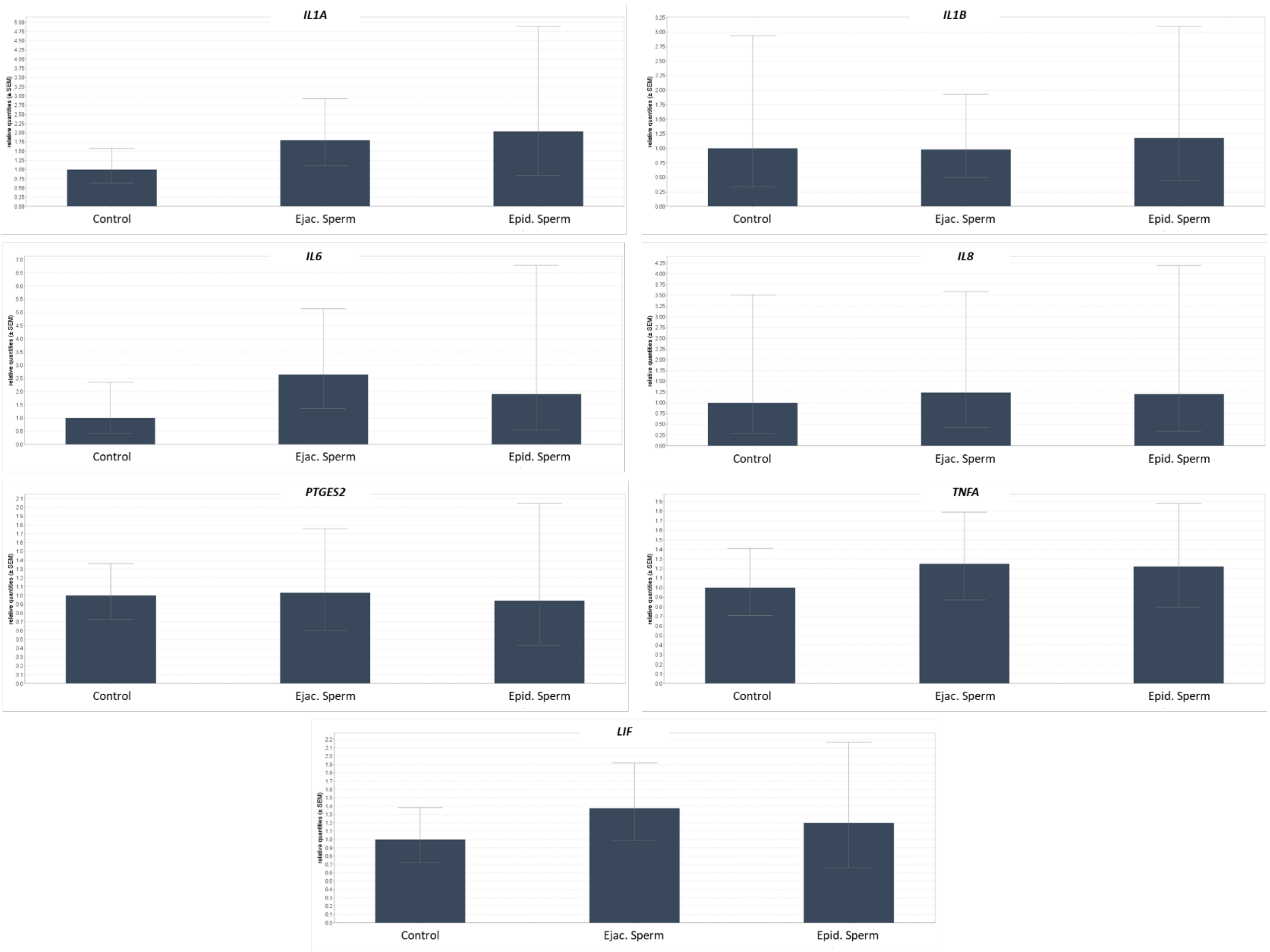

Supplementary Fig. 2 Uncropped electrophoresis gels of endometrial explants used to produce Fig. 2B and C. **A)** Lanes 1-9 (C1.1 to C1.9) correspond to: control, 1% bull seminal plasma (SP), 5% bull SP, 15% bull SP, 25% bull SP, 1% stallion SP, 5% stallion SP, 15% stallion SP, and 25% stallion SP. Explants C1.1 to C1.9 were recovered from the same heifer. **B)** Lanes 1-9 (M2.1 to M2.9) correspond to: control, 1% bull SP, 5% bull SP, 15% bull SP, 25% bull SP, 1% stallion SP, 5% stallion SP, 15% stallion SP, and 25% stallion SP. Explants M1.1 to M1.9 were recovered from the same mare.

A **B**

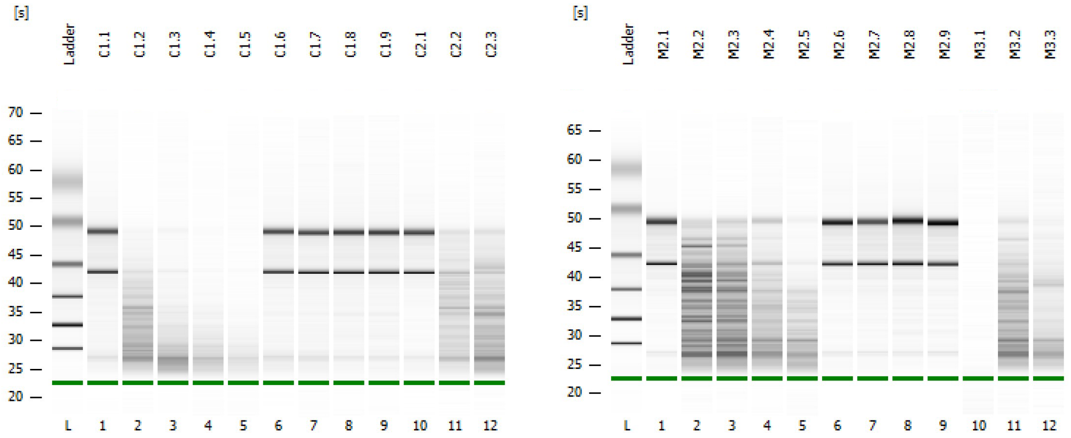

Supplementary Fig. 3 Uncropped electrophoresis gel of endometrial explants used to produce Fig. 3B. Lanes 1-7 (1.1 to 1.7) correspond to: control, 1% seminal plasma (SP) + inactivator reagent incubated at 60 ºC, 1% SP incubated at 60ºC, 1% SP+ inactivator reagent incubated at 45 ºC, 1% SP incubated at 45 º, 1% SP+ inactivator reagent incubated at 39 ºC, 1% SP incubated at 39 ºC. Explants 1.1 to 1.7 were recovered from the same heifer.

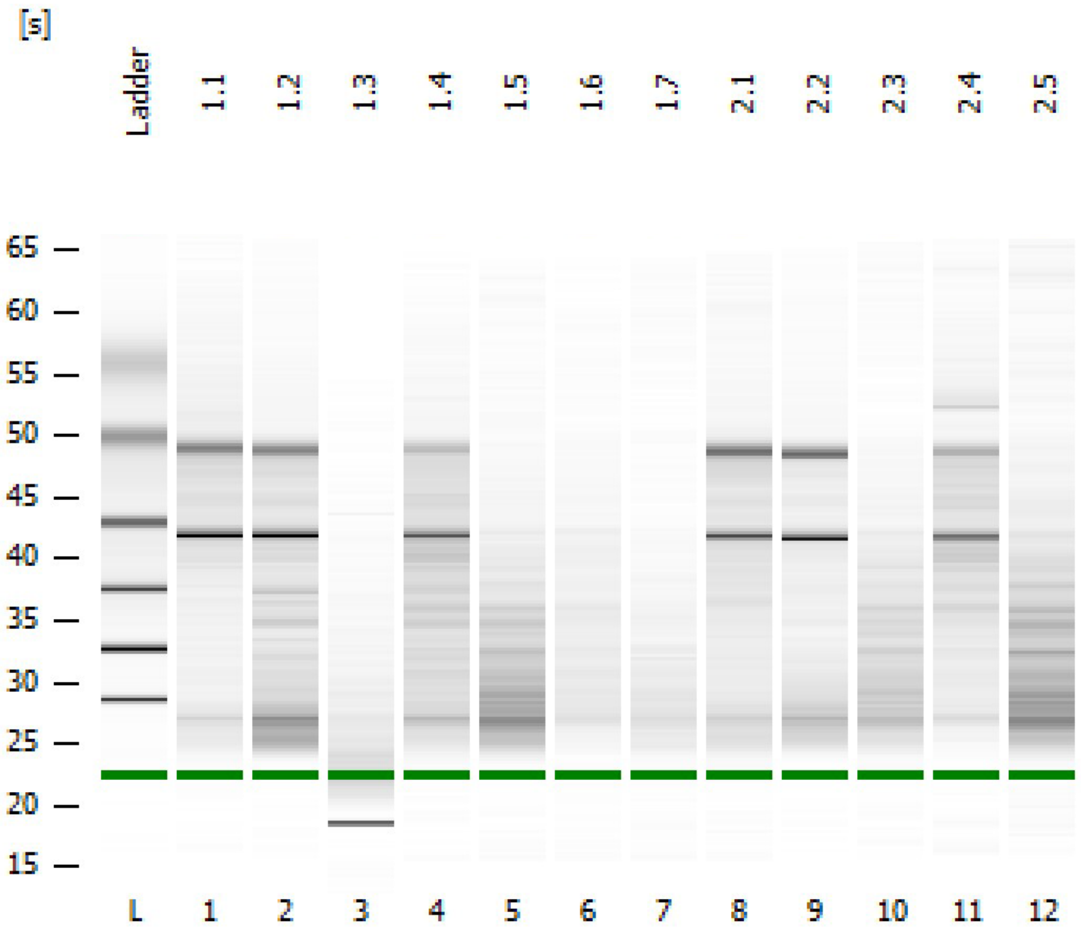

Supplementary Fig. 4 Uncropped electrophoresis gel of endometrial explants used to produce Fig. 4B and Fig. 5B. **A)** Lanes 3-12 (3.1 to 3.10) correspond to: vagina control, vagina + 1% seminal plasma (SP), vagina + 5% SP, vagina + 15% SP, vagina + 25% SP, cervix control, cervix +1% SP, cervix + 5% SP, cervix + 15% SP, cervix + 25% SP. **B)** Lanes 1 to 9 (3.11 to 3.19) correspond to: endometrium control, endometrium + 1% SP collected by artificial vagina (AV), endometrium + 5% SP AV, endometrium + 15% SP AV, endometrium + 25% SP AV, endometrium + 1% SP collected by electroejaculation (EE), endometrium + 5% SP EE, endometrium + 15% SP EE, endometrium + 25% EE. Note that explants 3.1 to 3.10 (A) and 3.11 to 3.19 (B) were recovered from the same heifer.

A

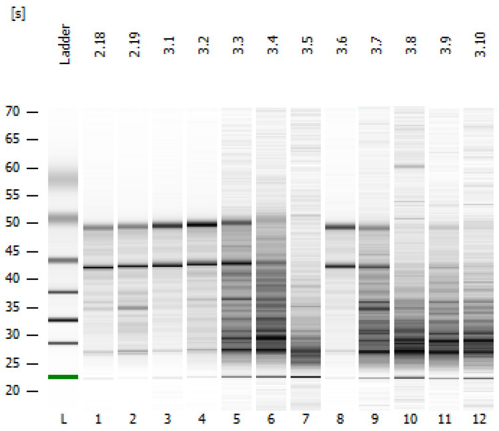

B

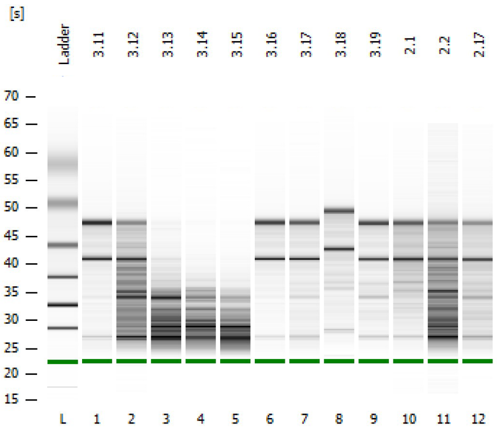

Supplement: Supplementary file 1 — Dataset 1 [file 41598_2019_51413_MOESM1_ESM.pdf]
